# Accelerated intermittent theta burst stimulation for pharmacological treatment‐resistant bipolar depression: Protocol for double‐blind, randomized, sham‐controlled trial

**DOI:** 10.1002/pcn5.70064

**Published:** 2025-02-10

**Authors:** Taro Kishi, Kenji Sakuma, Shun Hamanaka, Yasufumi Nishii, Kosei Esaki, Yueren Zhao, Yuki Matsuda, Shinsuke Kito, Nakao Iwata

**Affiliations:** ^1^ Department of Psychiatry Fujita Health University School of Medicine Toyoake Aichi Japan; ^2^ Department of Development and Education of Clinical Research Fujita Health University School of Medicine Toyoake Aichi Japan; ^3^ Department of Psychiatry Jikei University School of Medicine Minato‐ku Tokyo Japan

**Keywords:** accelerated intermittent theta burst stimulation, bipolar depression, double‐blind, efficacy, randomized sham‐controlled trial, safety

## Abstract

**Background:**

With 30%–50% of people with bipolar depression (BDep) not responding to multiple pharmacological treatments, alternative therapies are needed. Accelerated intermittent theta burst stimulation (aiTBS) over the left dorsolateral prefrontal cortex (L‐DLPFC) has been employed for individuals with pharmacological treatment‐resistant major depressive disorder (TR‐MDD). Imaging studies have revealed reduced regional activity of the L‐DLPFC for both TR‐MDD and pharmacological treatment‐resistant BDep (TR‐BDep), suggesting that aiTBS over the L‐DLPFC may be beneficial for people with TR‐BDep.

**Methods:**

A 6‐week, double‐blind, sham‐controlled, randomized trial will be conducted to compare the efficacy and safety of aiTBS to the L‐DLPFC in people with TR‐BDep (jRCTs042240019). Fifty iTBS sessions (1800 pulses/session) will be delivered in 10 daily sessions over 5 consecutive days at 90% resting motor threshold. This aiTBS protocol is termed as Fujita Neuromodulation Therapy for Bipolar Depression (FNT‐BD). Twenty‐two participants (both sexes, aged 18–64 years) with TR‐BDep (DSM‐5‐TR, Type I) will be recruited. The response rate at any given week of follow‐up will be the primary efficacy outcome, defined as a reduction of ≥50% in the Montgomery Åsberg Depression Rating Scale (MADRS) score. Other outcomes will include MADRS score changes, remission rate (10 ≥ MADRS score), Clinical Global Impression‐Improvement score, Clinical Global Impression‐Severity score, discontinuation rate, and incidence of individual adverse events.

**Results:**

We anticipate that individuals who receive the aiTBS treatment show significant improvement in depressing symptoms compared to those receiving sham treatment.

**Conclusions:**

This study will provide valuable evidence for both patients with TR‐BDep and clinicians.

## Graphical abstract

A 6‐week, double‐blind, sham‐controlled, randomized trial will be conducted to explore the efficacy and safety of accelerated intermittent theta burst stimulation over the left dorsolateral prefrontal cortex in people with pharmacological treatment‐resistant bipolar depression. This study will provide valuable evidence for people with pharmacological treatment‐resistant bipolar depression and for clinicians.

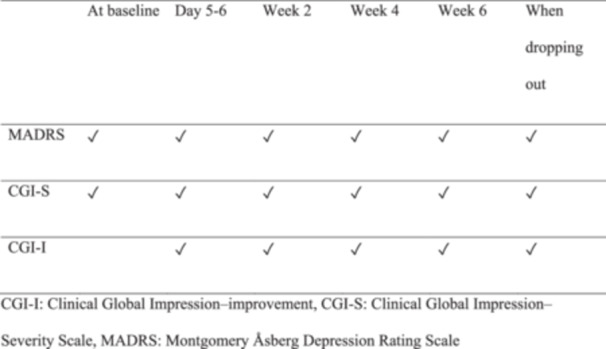

## INTRODUCTION

Bipolar disorder (BD) is a common and serious mental illness that affects approximately 1% of the population.^1^, ^2^ Individuals with BD frequently exhibit mania/hypomania or depression, which can cause social and occupational disability.^2^ A review of suicide cases in people with BD demonstrated that the risk of suicide is 20–30‐fold higher than in the general population.^3^ According to the *Diagnostic and Statistical Manual of Mental Disorders*, Fifth Edition, Text Revision (DSM‐5‐TR),^4^ bipolar I disorder can be established with the occurrence of a manic episode alone; however, the diagnosis of bipolar II disorder requires at least one distinct episode of hypomania and one distinct episode of major depression.

For both the acute and maintenance phases, including mania and depression, the primary treatment is pharmacological treatment using mood stabilizers (MSs) and second‐generation antipsychotics (SGAs).^2^, ^5^, ^6^ A recent network meta‐analysis found that olanzapine plus fluoxetine, quetiapine, olanzapine, lurasidone, lumateperone, cariprazine, and lamotrigine were more effective than placebo in reducing depressive symptoms, with moderate confidence in the evidence.^7^ However, 30%–50% of individuals with bipolar depression (BDep) fail to respond to multiple pharmacological treatments.^8^, ^9^, ^10^, ^11^ Thus, a novel treatment for these people is warranted.

Recently, repetitive transcranial magnetic stimulation (rTMS) has gained worldwide attention as a treatment option for various neurological and psychiatric conditions. The US Food and Drug Administration approved rTMS, a noninvasive therapeutic brain‐stimulation technique, for modulating the regional excitability of the human brain for pharmacological treatment‐resistant major depressive disorder (TR‐MDD), obsessive–compulsive disorder, and smoking cessation.^12^ rTMS directed at the left‐dorsolateral prefrontal cortex (L‐DLPFC) increases L‐DLPFC activity, which is underactive in patients with TR‐MDD and enhances therapeutic connections with the anterior cingulate and amygdala, both of which play vital roles in coordinating stress responses.^13^, ^14^, ^15^ Intermittent theta burst stimulation (iTBS) over the L‐DLPFC (iTBS [L‐DLPFC]) is a novel noninvasive therapeutic brain‐stimulation technique for TR‐MDD.^12^ Generally, iTBS is delivered more rapidly than traditional high‐frequency rTMS (HF‐rTMS).^16^, ^17^ A recent randomized controlled trial (RCT) elucidated that iTBS (L‐DLPFC) provided comparable effects on TR‐MDD to HF‐rTMS over the L‐DLPFC (HF‐rTMS [L‐DLPFC]),^18^ suggesting that iTBS (L‐DLPFC) may be a more practical and potentially more effective therapeutic modality. Our previous pairwise meta‐analysis also showed that iTBS (L‐DLPFC) and HF‐rTMS (L‐DLPFC) show no significant differences in efficacy, acceptability, and safety profiles.^17^

In both people with TR‐MDD and those with pharmacological treatment‐resistant BDep (TR‐BDep), imaging studies demonstrated a reduction in the regional activity of the L‐DLPFC.^19^, ^20^ Evidence also suggests that electroconvulsive therapy, another neuromodulation treatment, is effective for both TR‐MDD and TR‐BDep.^21^ In fact, our meta‐analysis indicated that rTMS was effective for both TR‐MDD and TR‐BDep.^22^ Furthermore, our meta‐regression analysis demonstrated no significant differences in the magnitude of the antidepressant effect of iTBS (L‐DLPFC) between people with TR‐MDD and those with TR‐BDep.^22^ However, the effect sizes of the current iTBS (L‐DLPFC) protocols on people with TR‐BDep were small.^22^ Recently, accelerated intermittent theta burst stimulation (aiTBS) over the L‐DLPFC (aiTBS [L‐DLPFC]), also known as Stanford Neuromodulation Therapy (SNT), has been demonstrated to be more effective for people with TR‐MDD.^23^ SNT utilizes resting‐state functional connectivity magnetic resonance imaging (MRI) to individually identify and target the region of the L‐DLPFC that is most anticorrelated with the subgenual anterior cingulate cortex in each participant.^23^ This protocol comprises 10 iTBS (L‐DLPFC) sessions that are delivered daily, for a total of 18,000 pulses per day on 5 consecutive days.^23^ An RCT of SNT found that the aiTBS (L‐DLPFC) outperformed sham in improving depressive symptoms, with a large effect size (Cohen's *d* > 0.8).^24^ We propose that developing an aiTBS (L‐DLPFC) protocol, such as SNT, for people with TR‐BDep is necessary.

## METHODS

### Study design

A 6‐week, double‐blind, sham‐controlled, randomized trial to compare the efficacy and safety of aiTBS (L‐DLPFC) in people with TR‐BDep will be conducted. The study has been registered with Japan Registry for Clinical Trials (jRCT) under the identifier jRCTs042240019. The study has been ongoing at the Fujita Health University Hospital (Toyoake) since September 2024 and for completion on March 31, 2027.

### Participants

Beginning in September 2024, a total of 22 eligible participants (males and females aged 18–64 years) will be recruited from outpatients or inpatients with TR‐BDep at Fujita Health University Hospital. A recent review reported that cognitive dysfunction occurs frequently in elderly adults with BD.^25^ A higher dementia prevalence is found in BD compared with healthy peers and other psychiatric populations.^25^ Therefore, our study will exclude elderly adults with BD. Every potential participant who consents will be invited to participate in the study. Full inclusion and exclusion criteria are summarized in Table 1.

**Table 1:** Inclusion and exclusion criteria.

*Inclusion criteria*
1. Individuals will be given a detailed description of the clinical trial, and all participants and their guardians will provide written informed consent. 2. Age: ≥18 years old and <65 years old at baseline 3. People who will meet the DSM‐5‐TR criteria for bipolar depression (Type I) 4. Individuals who will be diagnosed with pharmacological treatment‐resistant^a^ bipolar depression 5. People who will have a HAM‐D17 total score of 20 or higher at baseline
*Exclusion criteria*
1. Individuals with metal implants or devices near the stimulation site (e.g., cochlear implants, surgical clips with magnetic properties, or neurostimulators, such as deep brain stimulation or vagus nerve stimulation), individuals with a cardiac pacemaker 2. Individuals with metal implants or devices that are not near the stimulation site (e.g., implanted medication pumps), titanium products in their heads, magnetic dentures/implants 3. Individuals with a history of seizures, a history of intracranial lesions at risk of seizures, individuals taking drugs that reduce seizure threshold (methylphenidate or ketamine), individuals with alcohol/caffeine/stimulants abuse or withdrawal symptoms, pregnant individuals, and individuals with severe physical disease 4. Individuals who have received rTMS during their current depressive episode 5. Persons diagnosed with dementia, organic or symptomatic mood disorder 6. Individuals with unimproved depressive symptoms due to inadequate adherence to pharmacological treatment 7. Individuals diagnosed with substance‐related or medication‐induced mood disorders 8. Individuals who will answer “yes” to Question 1 (Do you have the intention to carry out a suicidal act, although you do not have a specific plan to do so?) or Question 2 (Do you have a specific plan and intention to carry out a suicidal act?) at baseline^b^ 9. Individuals deemed inappropriate by researchers

^a^ The definition is that an individual who will not respond to at least two mood stabilizers or second‐generation antipsychotics (valproate, lamotrigine, quetiapine extended‐release, lurasidone, or olanzapine that have been reported to have efficacy for bipolar depression by a network meta‐analysis^7^) with a sufficient dose (within the doses approved in Japan [PMDA]) for 2 weeks or more (CGI‐S score ≥4, we will confirm the result from their medical records). If the patients are receiving combination therapy with two or more of these drugs, we will choose the candidate drug with the highest DDD‐equivalent value among the drugs. If two or more drugs used the same DDD‐equivalent value during the same period, we will select one of these drugs to evaluate the definition.

DDD: https://www.who.int/tools/atc-ddd-toolkit/about-ddd

PMDA: https://www.pmda.go.jp/english/index.html

^b^ The questions referred to the Columbia‐Suicide Severity Rating Scale.^26^

Abbreviations: CGI‐S, Clinical Global Impression‐Severity Scale; DDD, defined daily dose; DSM‐5‐TR, *Diagnostic and Statistical Manual of Mental Disorders*‐Fifth Edition‐Text Revision; HAM‐D17, 17‐item Hamilton Rating Scale for Depression; PMDA, Pharmaceuticals and Medical Devices Agency; rTMS: repetitive transcranial magnetic stimulation.

TR‐BDep is defined as an individual who will not respond to at least two MSs or SGAs (valproate, lamotrigine, quetiapine extended‐release, lurasidone, or olanzapine that are reported to have efficacy for BDep by a network meta‐analysis^7^) with a sufficient dose (within the doses approved in Japan^27^) for 2 weeks or more (Clinical Global Impression‐Severity [CGI‐S]^28^ score ≥4, we will validate the result from their medical records). If patients are receiving combination therapy with two or more of these drugs, we will select the candidate drug with the highest defined daily dose (DDD),^29^ with an equivalent value among the drugs. If two or more drugs used the same DDD‐equivalent value during the same period, we will use one of them to evaluate the definition. Lithium is one of the key drugs for the treatment of BD^6^; however, recent network meta‐analysis showed that lithium did not improve depressive symptoms in people with BDep.^7^ Therefore, we did not include lithium as a candidate drug for nonresponse. Several diagnostic tests showed that patients who did not show minimal improvement by Week 2 of pharmacological treatment were unlikely to respond later and might benefit from a treatment change.^30^, ^31^ Therefore, the observation period for drug treatment response was set to a minimum of 2 weeks. Antipsychotics, MSs, antidepressants, anxiolytics, and/or sleeping pills will be permitted with doses maintained constantly throughout the period of randomized treatment.

### Primary and secondary outcome measures

The response rate at any given week of follow‐up is the primary efficacy outcome. The response will be defined as a reduction of ≥50% in the Montgomery Åsberg Depression Rating Scale (MADRS)^32^ score. Secondary efficacy outcomes include response rate at 5–7 days, Week 2, 4, and 6; changes in the MADRS score at 5–7 days, Week 2, 4, and 6; remission rate (10 ≥ MADRS score) at 5–7 days, Week 2, 4, and 6; Clinical Global Impression‐Improvement^28^ score at 5–7 days, Week 2, 4, and 6; CGI‐S for mania^28^ score at 5–7 days, Week 2, 4, and 6; and CGI‐S for depression^28^ score at 5–7 days, Week 2, 4, and 6. The definitions of “response” and “remission” in our study are the same as that of the SNT study.^23^, ^24^ Table 2 illustrates the schedule of these psychometric assessments. Other outcomes include the discontinuation rate and the incidence of individual adverse events.

**Table 2:** The SPIRIT schedule of these psychometric assessments.

	At baseline	Days 5–6	Week 2	Week 4	Week 6	When dropping out
MADRS	✔	✔	✔	✔	✔	✔
CGI‐S	✔	✔	✔	✔	✔	✔
CGI‐I		✔	✔	✔	✔	✔

Abbreviations: CGI‐I, Clinical Global Impression‐Improvement Scale; CGI‐S, Clinical Global Impression‐Severity Scale; MADRS, Montgomery Åsberg Depression Rating Scale.

### Intervention

Our study protocol basically follows that of the SNT study.^23^ A MagVenture MagPro R30 (MagVenture A/S) system and a double‐sided Cool‐B70 A/P coil will be used to deliver sessions of iTBS; 60 cycles of 10 bursts of three pulses at 50 Hz were delivered in 2‐s trains (5 Hz) with an 8‐s intertrain interval. Ten sessions will be applied per day (18,000 pulses/day) for 5 consecutive days (90,000 pulses in total). We will use BeamF3 for coil localization/targeting method.^33^ Recently, Sheline and colleagues reported that aiTBS (L‐DLPFC) similar to SNT protocol improved the depressive symptoms in patients with TR‐BDep.^34^ The resting motor threshold (RMT) of Sheline's study was 90%.^34^ Therefore, we decided that stimulation will be delivered at 90% of the RMT in our study. First, beginning at 40% of maximal stimulator output, the approximate RMT will be determined by moving the coil and adjusting the power level until exactly three right thumb twitches during five consecutive pulses are produced. In this step, the stimulus intensity will be gradually increased in steps of 5% of the maximal stimulator output. Second, the almost RMT will be determined by decreasing the stimulus intensity in 1% steps, as the minimum intensity (as a percentage of the maximal stimulator output) that will produce exactly two right thumb twitches during five consecutive pulses. Finally, the RMT will be determined as the almost RMT plus 1% stimulus intensity. We also referred to the Japanese rTMS text^35^ and a previous systematic review^36^ for these methods. A specially designed rTMS coil with both an active and a sham inductor head including an identical external appearance will be employed to ensure blindness during treatment. Sham treatment will include a simultaneous electric pulse to mimic aiTBS sensation. Participants will be seated in a designated waiting area that will not be shared by study staff or other patients in between treatments. This will be conducted to limit interaction time with study staff and to avoid a group effect. All iTBS sessions will be performed in the Department of Psychiatry at Fujita Health University Hospital.

Because we want to develop an aiTBS protocol that is more convenient in clinical practice than SNT,^23^ the SNT protocol was modified in the following manner: (1) For coil localization/targeting method, SNT uses MRI. We will use BeamF3^33^ instead of MRI because our meta‐regression analysis demonstrated no differences in the effect size of iTBS (L‐DLPFC) for the improvement of depressive symptoms between studies using MRI and those that did not.^22^ Sheline's study also used MRI for determining the stimulation target.^34^ (2) For intersession interval, the intersession interval of SNT and Sheline's study was 50 min. The intersession interval of our study will be 30–50 min because our meta‐regression analysis revealed that length of intersession interval was unrelated to the magnitude of effect size of iTBS (L‐DLPFC) for the improvement of depressive symptoms.^22^ We refer to this aiTBS protocol as Fujita Neuromodulation Therapy for Bipolar Depression (FNT‐BD).

### Randomization and blinding

Eligible participants will be randomly assigned (1:1) to either aiTBS or a sham treatment using a computer‐generated list of random numbers. Randomization will be stratified by sex (men vs. women) and age (<46 or ≥46 years). The age threshold was established at 46 years because the median age of outpatients at our hospital who satisfied the TR‐BDep criteria as defined in this study was 46 years. The allocation sequences will be added to the Research Electronic Data Capture (REDCap) electronic database.^37^, ^38^ An independent researcher from the Center for Clinical Trial and Research Support, Fujita Health University School of Medicine will conduct the randomization and allocation. The sequence will be unknown to all participants, caregivers, clinical assessors, treatment providers, and other study personnel.

All participants, caregivers, clinical assessors, treatment providers, and other study personnel will be unaware of their treatment assignments. Clinical assessors and treatment providers will be separate entities. Participants will be instructed not to discuss stimulation sensations with the study personnel. Each iTBS session will use the same stimulation coil, with no indication of active or sham orientation. Hence, all participants, clinical assessors, treatment providers, and other study personnel should be unable to infer that sham treatment will be used, that is, they should be unaware of the stimulation condition.

### Sample size calculation

According to the SNT study data (response rate at any week of follow‐up in the aiTBS group and sham group was 85.7% and 26.7%, respectively),^24^ allocating 11 participants to each arm will ensure ≥80% power to detect the superiority of the aiTBS compared to the sham group regarding the primary outcome. A chi‐square test and a two‐tailed test were used to determine the sample size with a statistical significance of 0.05. Because approximately 7% of randomized participants dropped out of the SNT study,^24^ we factored this information into our sample size calculation.

### Statistical analysis

Descriptive statistics will be computed for all variables of interest. Continuous measures, like age, will be summarized using means and standard deviations, while categorical measures will be summarized with counts and percentages. The data will be analyzed on a full analysis set, with endpoint analysis using the last‐observation‐carried forward method. The full analysis set will include all patients who agree to provide a baseline and at least one postbaseline data measurement. Response and remission rates will be evaluated using a *χ* ^2^ test. In Week 6, the MADRS score will be calculated using generalized linear mixed models. All statistical tests will be two‐sided, with significance defined as *p* < 0.05.

### Ethics and dissemination

This research protocol was peer‐reviewed and approved by the Ethical Review Board of Fujita Health University (CR24‐028). This research will be conducted in accordance with the Declaration of Helsinki and the Ethical Guidelines for Medical and Biological Research Involving Human Subjects as certified by the Japanese Ministry of Education, Culture, Sports, Science and Technology; the Ministry of Health, Labour, and Welfare; and the Ministry of Economy, Trade and Industry. Any protocol amendments (changes to eligibility criteria, outcomes, and analyses) that are necessary will be communicated and modified in the relevant parties. All required documents, including the study protocol, informed consent form, participant information leaflet, and any other documents have been reviewed and approved by the Ethics Review Committee of Fujita Health University on August 14, 2024.

Following screening, we will invite potentially eligible patients to join our study. All procedures involved, including the potential benefits and risks, will be discussed in detail. They will also be informed that participation in the study is entirely voluntary, and their treatment will not be affected if they decline to participate. All questions and concerns will be addressed in detail. Participants who satisfy the inclusion but not the exclusion criteria can only be recruited after providing written informed consent. Every participant's personal information will be strictly confidential. Serial numbers will be used instead of real names when analyzing data.

We acknowledge funding from the Japan Agency for Medical Research and Development (24dk0307129h0001). The funding source will play no role in the study's design and execution, data collection, management, analysis, interpretation, manuscript preparation, review, approval, or decision to submit the manuscript for publication. For each visit, participants in this study will receive a monetary compensation of 10,000 Japanese yen. The study data will be collected and managed using REDCap electronic data capture tools hosted at Fujita Health University School of Medicine.^37^, ^38^ REDCap is a secure, web‐based software platform designed to support data capture for research studies. It includes (1) an intuitive interface for validated data capture; (2) audit trails for tracking data manipulation and export procedures; (3) automated export procedures for seamless data downloads to common statistical packages; and (4) data integration and interoperability procedures with external sources. Access to study data in REDCap will be restricted to study team members who have authenticated using Fujita Health University credentials.

Data is handled, computerized, and stored in accordance with the Clinical Trials Act of 2017. Paper copies of trial‐related documentation are annotated, signed, and dated before being filed in the electronic medical records. The Principal Investigator is responsible for the overall quality and retention of trial data. All trial data are retained following the most recent Directive on GCP (2005/28/EC) and local policy.

Staff collect clinical and safety data for trial participants, which are then recorded in the electronic case report form (eCRF) of the clinical data management system REDCap and password‐protected trial spreadsheets. The eCRFs and trial spreadsheets utilize unique trial identifier numbers to identify participants. A Data Monitoring Committee evaluates patient safety in a clinical trial of an investigational intervention through periodic review of adverse events and clinical safety assessments. Posttrial care of the study participants is ensured by the possibility of further treatment in the standard care setting.

Study results will be disseminated through publication in international peer‐reviewed journals and conference presentations to mental health professionals.

## DISCUSSION

A recent network meta‐analysis elucidated that olanzapine plus fluoxetine, quetiapine, olanzapine, lurasidone, lumateperone, cariprazine, and lamotrigine were more effective than placebo at reducing depressive symptoms,^7^ although 30%–50% of individuals with BDep are refractory to pharmacological treatments.^8^, ^9^, ^10^, ^11^ Thus, we believe that developing a novel treatment for these people is warranted. The proposed study will be the first to use an RCT to explore the effects of a high‐dose iTBS (L‐DLPFC) protocol on TR‐BDep. We anticipate that people receiving our aiTBS (L‐DLPFC) treatment will experience significant improvements in depressive symptoms compared to those receiving sham treatment. According to the SNT study, the aiTBS (L‐DLPFC) protocol was well tolerated by the participants.^24^ This study will provide important evidence for TR‐BDep patients and clinicians.

Our study protocol has several limitations. First, the period during which response to the drug is evaluated may be short (minimum 2 weeks). Second, our study allows the use of concomitant medications. Because benzodiazepine administration may inhibit rTMS response, whereas psychostimulant use may be associated with a greater rTMS response,^39^, ^40^ the medications might influence the study results. Third, the SNT study is often stimulated at approximately 110% of the RMT after correction.^23^, ^24^ Because the protocol in our study does not use individual navigation for each participant, depth correction might be difficult to implement, raising concerns that the stimulation intensity may be low. Fourth, the range of interval session in our study is 30–50 min, and the factor might be a confounding factor. We will report an association between factors that will be included in the statistical analysis as a variable and the results of our study in the next paper.

## AUTHOR CONTRIBUTIONS

Taro Kishi designed the study, obtained funding and ethics approval, wrote the study protocol, and registered the study at the clinical trials. Taro Kishi, Kenji Sakuma, Yasufumi Nishii, Shun Hamanaka, Yueren Zhao, Kosei Esaki, and Yuki Matsuda will contribute to recruiting participants, will organize the study procedures, will conduct assessments, and will carry out both interventions. Shinsuke Kito and Nakao Iwata will supervise the study procedures. All authors discussed and critically wrote the manuscript.

## CONFLICT OF INTEREST STATEMENT

All authors have no conflicts of interest to declare concerning this study. They also declare any potential competing interests that have arisen in the last 3 years. Professor Kishi has received speaker's honoraria from Eisai, Janssen, Meiji, Otsuka, Sumitomo, Takeda, Mitsubishi‐Tanabe, Kyowa, Yoshitomi, and Viatris and research grants from Eisai, JSPS KAKENHI (19K08082 and 23K06998), Japan Agency for Medical Research and Development (JP22dk0307107, JP22wm0525024, 23dk0307117h0001, 24dk0307129h0001, and 24dk0307129h0001), and the Japanese Ministry of Health, Labour, and Welfare (21GC1018). Dr. Sakuma has received speaker's honoraria from daiichisankyo, Eisai, Janssen, Kyowa, Meiji, Otsuka, Sumitomo, and Takeda and has received a Fujita Health University School of Medicine Research Grant for Early‐Career Scientists, Grant‐in‐Aid for Young Scientists (B)(19K17099), Grant‐in‐Aid for Scientific Research (C)(23K06998), and Japan Agency for Medical Research and Development (JP22dk0307107 and JP23dk0307122). Dr. Hamanaka has received speaker's honoraria from Meiji, Otsuka, and Sumitomo. Dr. Nishii has received speaker's honoraria from Meiji, Otsuka, and Sumitomo. Dr. Esaki has received speaker's honoraria from Sumitomo and Takeda. Dr. Zhao has nothing to disclose. Dr. Matsuda has received speaker's honoraria from Otsuka, Sumitomo, Takeda, Teijin, Lundbeck, and Viatris and research grants from Japan Agency for Medical Research and Development (24dk0307126h0001). Professor Kito received speaker's honoraria from Inter Reha, Lundbeck, Sumitomo, Otsuka, Takeda, Teijin, and Viatris; consultant fees from Teijin; and research grants from Teijin. Professor Iwata has received speaker's honoraria from Eisai, Janssen, Meiji, Otsuka, Sumitomo, Takeda, Mitsubishi‐Tanabe, and Viatris and research grants from Daiichi Sankyo, Eisai, Meiji, Otsuka, Sumitomo, Takeda, Tanabe‐Mitsubishi, Grant‐in‐Aid for Scientific Research (B)(22H03003), and Japan Agency for Medical Research and Development (JP22wm0425008). Prof. Iwata is the Editor‐in‐Chief of *Psychiatry and Clinical Neurosciences Reports* and a co‐author of this article. Prof. Iwata was excluded from editorial decision‐making related to the acceptance and publication of this article.

## ETHICS APPROVAL STATEMENT

The protocol for this research project has been approved by a suitably constituted Ethics Committee of the institution and it conforms to the provisions of the Declaration of Helsinki. Committee of Fujita Health University, Approval No. CR24‐028.

## PATIENT CONSENT STATEMENT

Following screening, we will invite potentially eligible patients to join our study. All procedures involved, as well as the potential benefits and risks, will be discussed in detail. They will also be informed that participation in the study is entirely voluntary, and their treatment will not be affected if they decline to participate. All questions and concerns will be addressed in detail. Participants who meet the inclusion but not the exclusion criteria can only be recruited after providing written informed consent. Every participant's personal information will be kept strictly confidential. Serial numbers will be used instead of real names when analyzing data.

## CLINICAL TRIAL REGISTRATION

The study has been registered with Japan Registry for Clinical Trials under the identifier jRCTs042240019.

## Data Availability

As our research dataset contains potentially sensitive information, we have ethical restrictions. Please contact the Institutional Review Board of the Ethics Committee when requesting data.

## References

[1] Alonso J , Petukhova M , Vilagut G , Chatterji S , Heeringa S , Üstün TB , et al. Days out of role due to common physical and mental conditions: results from the WHO World Mental Health surveys. Mol Psychiatry. 2011;16:1234–1246. 20938433 10.1038/mp.2010.101 PMC3223313

[2] Grande I , Berk M , Birmaher B , Vieta E . Bipolar disorder. Lancet. 2016;387:1561–1572. 26388529 10.1016/S0140-6736(15)00241-X

[3] Pompili M , Gonda X , Serafini G , Innamorati M , Sher L , Amore M , et al. Epidemiology of suicide in bipolar disorders: a systematic review of the literature. Bipolar Disord. 2013;15:457–490. 23755739 10.1111/bdi.12087

[4] APA . Diagnostic and statistical manual of mental disorders. 5th edition. Washington, DC: Text Revision American Psychiatric Association Publishing; 2022.

[5] Baldessarini RJ , Tondo L , Vázquez GH . Pharmacological treatment of adult bipolar disorder. Mol Psychiatry. 2019;24:198–217. 29679069 10.1038/s41380-018-0044-2

[6] Kato T , Ogasawara K , Motomura K , Kato M , Tanaka T , Takaesu Y , et al. Practice guidelines for bipolar disorder by the JSMD (Japanese Society of Mood Disorders). Psychiatry Clin Neurosci. 2024;78:633–645. 39194164 10.1111/pcn.13724 PMC11804931

[7] Yildiz A , Siafis S , Mavridis D , Vieta E , Leucht S . Comparative efficacy and tolerability of pharmacological interventions for acute bipolar depression in adults: a systematic review and network meta‐analysis. Lancet Psychiatry. 2023;10:693–705. 37595997 10.1016/S2215-0366(23)00199-2

[8] De Fruyt J , Deschepper E , Audenaert K , Constant E , Floris M , Pitchot W , et al. Second generation antipsychotics in the treatment of bipolar depression: a systematic review and meta‐analysis. J Psychopharmacol. 2012;26:603–617. 21940761 10.1177/0269881111408461

[9] Fountoulakis KN . Refractoriness in bipolar disorder: definitions and evidence‐based treatment. CNS Neurosci Ther. 2012;18:227–237. 22070611 10.1111/j.1755-5949.2011.00259.x PMC6493614

[10] Diaz AP , Fernandes BS , Quevedo J , Sanches M , Soares JC . Treatment‐resistant bipolar depression: concepts and challenges for novel interventions. Braz J Psychiatry. 2022;44:178–186. 34037084 10.1590/1516-4446-2020-1627 PMC9041963

[11] Elsayed OH , Ercis M , Pahwa M , Singh B . Treatment‐resistant bipolar depression: therapeutic trends, challenges and future directions. Neuropsychiatr Dis Treat. 2022;18:2927–2943. 36561896 10.2147/NDT.S273503 PMC9767030

[12] US Food and Drug Administration (FDA) . [cited 2025 Jan 6].

[13] Sultana T , Hasan MA , Kang X , Liou‐Johnson V , Adamson MM , Razi A . Neural mechanisms of emotional health in traumatic brain injury patients undergoing rTMS treatment. Mol Psychiatry. 2023;28:5150–5158. 37414927 10.1038/s41380-023-02159-z PMC11041778

[14] Grosshagauer S , Woletz M , Vasileiadi M , Linhardt D , Nohava L , Schuler AL , et al. Chronometric TMS‐fMRI of personalized left dorsolateral prefrontal target reveals state‐dependency of subgenual anterior cingulate cortex effects. Mol Psychiatry. 2024;29:2678–2688. 38532009 10.1038/s41380-024-02535-3 PMC11420068

[15] Satterthwaite TD , Cook PA , Bruce SE , Conway C , Mikkelsen E , Satchell E , et al. Dimensional depression severity in women with major depression and post‐traumatic stress disorder correlates with fronto‐amygdalar hypoconnectivty. Mol Psychiatry. 2016;21:894–902. 26416545 10.1038/mp.2015.149 PMC4840084

[16] Kishi T , Ikuta T , Sakuma K , et al Repetitive transcranial magnetic stimulation for bipolar depression: a systematic review and pairwise and network meta‐analysis. Mol Psychiatry. Jan 2024; Jan 29(1):39–42. 37020049 10.1038/s41380-023-02045-8 PMC11078724

[17] Kishi T , Sakuma K , Matsuda Y , Kito S , Iwata N . Intermittent theta burst stimulation vs. high‐frequency repetitive transcranial magnetic stimulation for major depressive disorder: a systematic review and meta‐analysis. Psychiatry Res. 2023;328:115452. 37657200 10.1016/j.psychres.2023.115452

[18] Blumberger DM , Vila‐Rodriguez F , Thorpe KE , Feffer K , Noda Y , Giacobbe P , et al. Effectiveness of theta burst versus high‐frequency repetitive transcranial magnetic stimulation in patients with depression (THREE‐D): a randomised non‐inferiority trial. Lancet. 2018;391:1683–1692. 29726344 10.1016/S0140-6736(18)30295-2

[19] Moebus L , Quirin M , Ehrlenspiel F . Cerebral asymmetry in bipolar disorders: a scoping review. Biol Psychol. 2023;179:108551. 37059217 10.1016/j.biopsycho.2023.108551

[20] Fitzgerald PB . An update on the clinical use of repetitive transcranial magnetic stimulation in the treatment of depression. J Affect Disord. 2020;276:90–103. 32697721 10.1016/j.jad.2020.06.067

[21] Bahji A , Hawken ER , Sepehry AA , Cabrera CA , Vazquez G . ECT beyond unipolar major depression: systematic review and meta‐analysis of electroconvulsive therapy in bipolar depression. Acta Psychiatr Scand. 2019;139:214–226. 30506992 10.1111/acps.12994

[22] Kishi T , Ikuta T , Sakuma K , Hatano M , Matsuda Y , Wilkening J , et al. Theta burst stimulation for depression: a systematic review and network and pairwise meta‐analysis. Mol Psychiatry. 2024;29:3893–3899. 38844532 10.1038/s41380-024-02630-5 PMC11609094

[23] Cole EJ , Stimpson KH , Bentzley BS , Gulser M , Cherian K , Tischler C , et al. Stanford accelerated intelligent neuromodulation therapy for treatment‐resistant depression. Am J Psychiatry. 2020;177:716–726. 32252538 10.1176/appi.ajp.2019.19070720

[24] Cole EJ , Phillips AL , Bentzley BS , Stimpson KH , Nejad R , Barmak F , et al. Stanford Neuromodulation Therapy (SNT): a double‐blind randomized controlled trial. Am J Psychiatry. 2022;179:132–141. 34711062 10.1176/appi.ajp.2021.20101429

[25] Beunders AJM , Orhan M , Dols A . Older age bipolar disorder. Curr Opin Psychiatry. 2023;36:397–404. 37458495 10.1097/YCO.0000000000000883 PMC10399956

[26] Posner K , Brown GK , Stanley B , Brent DA , Yershova KV , Oquendo MA , et al. The Columbia‐Suicide Severity Rating Scale: initial validity and internal consistency findings from three multisite studies with adolescents and adults. Am J Psychiatry. 2011;168:1266–77. 22193671 10.1176/appi.ajp.2011.10111704 PMC3893686

[27] Pharmaceuticals and Medical Devices Agency (PMDA ). [cited 2025 Jan 6]. Available from: https://www.pmda.go.jp/english/index.html

[28] Guy W . ECDEU assessment manual for psychopharmacology: US Department of Health, Education and Welfare Publication (ADM). Rockville, MD: National Institute of Mental Health; 1976. p. 76–338.

[29] WHO . Defined daily dose (DDD). [cited 2025 Jan 6]. https://www.who.int/tools/atc-ddd-toolkit/about-ddd

[30] Kishi T , Nakamura H , Kato T , Iwata N . A diagnostic test to examine early improvement as a predictor of later response to lurasidone in bipolar depression. Neuropsychopharmacol Rep. 2023;43:137–140. 36632763 10.1002/npr2.12319 PMC10009426

[31] Kemp DE , Ganocy SJ , Brecher M , Carlson BX , Edwards S , Eudicone JM , et al. Clinical value of early partial symptomatic improvement in the prediction of response and remission during short‐term treatment trials in 3369 subjects with bipolar I or II depression. J Affect Disord. 2011;130:171–179. 21071096 10.1016/j.jad.2010.10.026 PMC3073691

[32] Montgomery SA , Åsberg M . A new depression scale designed to be sensitive to change. Br J Psychiatry. 1979;134:382–389. 444788 10.1192/bjp.134.4.382

[33] Beam W , Borckardt JJ , Reeves ST , George MS . An efficient and accurate new method for locating the F3 position for prefrontal TMS applications. Brain Stimulat. 2009;2:50–54. 10.1016/j.brs.2008.09.006 PMC2882797 20539835

[34] Sheline YI , Makhoul W , Batzdorf AS , Nitchie FJ , Lynch KG , Cash R , et al. Accelerated intermittent theta‐burst stimulation and treatment‐refractory bipolar depression: a randomized clinical trial. JAMA Psychiatry. 2024;81:936–941. 38985492 10.1001/jamapsychiatry.2024.1787 PMC11238064

[35] Kito S . TMS for depression. Bunkyo‐ku, Tokyo, Japan: Kanehara & Co. Ltd; 2016.

[36] Cavaleri R , Schabrun SM , Chipchase LS . The number of stimuli required to reliably assess corticomotor excitability and primary motor cortical representations using transcranial magnetic stimulation (TMS): a systematic review and meta‐analysis. Syst Rev. 2017;6:48. 28264713 10.1186/s13643-017-0440-8 PMC5340029

[37] Harris PA , Taylor R , Minor BL , Elliott V , Fernandez M , O'Neal L , et al. The REDCap consortium: building an international community of software platform partners. J Biomed Inf. 2019;95:103208. 10.1016/j.jbi.2019.103208 PMC7254481 31078660

[38] Harris PA , Taylor R , Thielke R , Payne J , Gonzalez N , Conde JG . Research electronic data capture (REDCap)—a metadata‐driven methodology and workflow process for providing translational research informatics support. J Biomed Inf. 2009;42:377–381. 10.1016/j.jbi.2008.08.010 PMC2700030 18929686

[39] Hunter AM , Minzenberg MJ , Cook IA , Krantz DE , Levitt JG , Rotstein NM , et al. Concomitant medication use and clinical outcome of repetitive Transcranial Magnetic Stimulation (rTMS) treatment of major depressive disorder. Brain Behav. 2019;9:e01275. 30941915 10.1002/brb3.1275 PMC6520297

[40] Deppe M , Abdelnaim M , Hebel T , Kreuzer PM , Poeppl TB , Langguth B , et al. Concomitant lorazepam use and antidepressive efficacy of repetitive transcranial magnetic stimulation in a naturalistic setting. Eur Arch Psychiatry Clin Neurosci. 2021;271:61–67. 32648109 10.1007/s00406-020-01160-9 PMC7867521

